# Efficacy of povidone and propylene glycol–based artificial tears in the treatment of dry eye disease

**DOI:** 10.5935/0004-2749.2025-0153

**Published:** 2025-09-10

**Authors:** Laura Caldas dos Santos, Alléxya Affonso, Rubens Belfort Jr., Denise de Freitas

**Affiliations:** 1 Department of Ophthalmology and Visual Sciences, Escola Paulista de Medicina, Hospital São Paulo, Universidade Federal de São Paulo, São Paulo, SP, Brazil; 2 Instituto da Visão, Instituto Paulista de Estudos e Pesquisas em Oftalmologia, São Paulo, SP, Brazil

**Keywords:** Artificial tears, Dry eye disease, Tear-film stability, Propylene glycol, Povidone, Visual acuity, Surveys and questionnaires

## Abstract

**Purpose:**

This clinical study aimed to assess the effectiveness of microemulsion artificial tears containing povidone and propylene glycol in the management of dry eye disease. Secondary objectives included evaluating improvements in tear-film stability, measured by tear break-up time and corneal staining scores, along with the tolerability and safety of the formulation.

**Methods:**

This was a prospective, single-arm interventional study involving 30 participants (52 eyes) diagnosed with dry eye disease. Participants self-administered the investigational eye drops twice daily for 28 consecutive days. Primary and secondary outcomes included changes in the Ocular Surface Disease Index, tear break-up time, and corneal staining scores. Adverse events were documented throughout the study period.

**Results:**

Significant improvements in Ocular Surface Disease Index scores were observed, reflecting a reduction in dry eye disease symptoms. Tear break-up time increased notably between baseline and follow-up assessments, with the proportion of eyes exhibiting tear break-up time ≥10 s rising from 25.0% to 63.5%. Additionally, the percentage of eyes with a corneal staining score of zero improved from 23.1% to 69.2%. Conjunctival staining also decreased, with the proportion of eyes with scores of 2 and 3 dropping from 11.5% to 3.8% and 5.8% to 0%, respectively.

**Conclusions:**

The findings suggest that povidone and propylene glycol-based artificial tears significantly enhance tear-film stability and alleviate symptoms in patients with mild to moderate dry eye disease, with minimal adverse effects. This formulation represents a safe and effective short-term treatment option for dry eye disease management.

## INTRODUCTION

Tear-film instability is a hallmark of dry eye disease (DED), contributing to symptoms such as dryness, irritation, and fluctuating or blurred vision^(1^-^3)^. A stable tear film is essential for maintaining optical quality and ocular surface health, as it provides a smooth refractive interface, protects against environmental insults, and reduces friction during blinking^(4^-^6)^. Disruption of this stability not only impairs visual clarity but also compromises the protective and lubricating functions of the ocular surface.

Effective management of DED requires therapeutic approaches that address both aqueous tear deficiency and dysfunction of the lipid layer^(7)^. Artificial tear formulations that target these components can relieve symptoms, restore tear-film homeostasis, and prevent further damage to the ocular surface^(8)^. Lipid-containing eye drops, including those with mineral oil, castor oil, or phospholipids, mimic the natural lipid layer, reduce tear evaporation, and enhance tear-film stability, thereby providing prolonged symptomatic relief^(9)^.

Povidone, a synthetic polymer with mucoadhesive properties, helps retain moisture on the ocular surface, while propylene glycol, a common humectant, aids in maintaining hydration. Both agents contribute to the alleviation of DED symptoms by enhancing the mucoa queous layer.

A novel microemulsion formulation of artificial tears (Rohto Dry Aid; ROHTO Pharmaceutical Co., Ltd., Ikuno-ku, Osaka, Japan) contains povidone and propylene glycol, along with lipid-replenishing components such as sesame oil, polyoxyethylene castor oil, and polyoxyl stearate. These ingredients are designed to replicate the properties of natural tears and stabilize the tear film^(10)^. In addition, the formulation includes poloxamer, which improves water retention and reduces ocular surface friction^(10)^. The present study aimed to evaluate the clinical efficacy and safety of this microemulsion-based lubricant when administered twice daily over a 28-day period in patients with DED. The primary objective was to assess improvements in tear-film integrity, as measured by tear break-up time (TBUT) and corneal staining with vital dyes. Secondary objectives included evaluating the safety profile and recording any adverse events associated with the treatment.

## METHODS

The study protocol and informed consent form were approved by the Research Ethics Committee of the Centro Universitário Faculdade de Medicina do ABC (CAAE: 65207522.0.0000.008).This prospective, single-arm, interventional study was conducted at the *Instituto Paulista de Estudos e Pesquisas em Oftalmologia* in São Paulo, Brazil. Eligible participants included patients diagnosed with aqueous-deficient, evaporative, or mixed-type DED. Those who met the predefined inclusion and exclusion criteria were invited to participate.

The study consisted of three scheduled visits: two in-person assessments and one remote consultation. The first visit (Visit 1-Day 0 [D0]) involved screening and baseline evaluation. The second visit (Visit 2-Day 14 [D14]) was conducted via teleconsultation and involved adherence assessment and symptom monitoring. The final in-person visit (Visit 3-Day 28 [D28]) included posttreatment evaluations.

Participants were instructed to instill one drop of the study medication-Rohto Dry Aid (ROHTO Pharmaceutical Co., Ltd., Ikuno-ku, Osaka, Japan)-into each eye twice daily (morning and evening) for 28 consecutive days. This microemulsion formulation, containing povidone and propylene glycol, was administered topically. If a dose was missed, patients were instructed to apply it as soon as possible and record the incident in a provided treatment diary. Participants who discontinued treatment for any reason were withdrawn from the study.

At Visit 1 (screening), the following data were collected: demographic information; ocular and systemic medical histories; and details of current ocular and systemic medications. Comprehensive ophthalmic examinations included best-corrected visual acuity (VA) (BCVA), slit-lamp biomicroscopy, tear-film break-up time (TFBUT), corneal and conjunctival staining with fluorescein and lissamine green, meibomian gland function assessment, intraocular pressure (IOP) measurement, fundus examination, and the Schirmer I test. Patient-reported outcomes were assessed using the Ocular Surface Disease Index (OSDI), the EuroQol 5-Dimensions 5-Levels (EQ-5D-5L) questionnaire, and an adverse effects questionnaire. Urine pregnancy testing was conducted for participants of childbearing potential.The EQ-5D-5L (Brazilian Portuguese version 3.1; interviewer-assisted version 1.2 when applicable), a validated instrument from the EuroQol Research Foundation, was employed both at baseline and during follow-up.

At Visit 2 (D14), a teleconsultation was conducted to reassess OSDI and EQ-5D-5L scores, monitor adherence, and evaluate adverse effects. Pain intensity was also assessed using the following scale: 0-2 (*no pain*), 3-7 (*moderate pain*), and 8-10 (*severe pain*).

At Visit 3 (D28), participants underwent a second in-person evaluation. Changes in health status and medication use were recorded. The same clinical examinations from Visit 1 were repeated, including BCVA, slit-lamp biomicroscopy, TFBUT, ocular surface staining, meibomian gland evaluation, IOP measurement, and fundus examination. OSDI and EQ-5D-5L questionnaires were readministered, and pain was measured using a visual analog scale. Any reported adverse effects were documented and discussed with the patient.

### Inclusion criteria

Eligible participants were required to be 18 yr of age or older with a documented history of DED lasting at least 6 months. A TFBUT of ≤5 s in at least one eye and the presence of dry eye symptoms, as indicated by an OSDI score >13, were mandatory. Additionally, participants were required to exhibit at least one of the following clinical signs in at least one eye: (1) a Schirmer I test result of ≤9 mm or (2) a meibomian gland secretion quality and expressibility score of ≥1 (on a scale from 0 to 3). Best-corrected VA had to be ≥20/80 in both eyes. All participants were classified as having mild to moderate DED and were instructed to discontinue the use of artificial tear supplements prior to participation.

### Exclusion criteria

Exclusion criteria included a known hypersensitivity to the investigational product or any of its components. Additional exclusion conditions were as follows: use of any topical ocular medication preserved with benzalkonium chloride within one month prior to the screening visit; the presence of ocular abnormalities (e.g., eyelid malformations, corneal disease, ocular surface metaplasia, or recurrent corneal erosion); corneal neovascularization; a history of herpes simplex or zoster keratitis; active ocular infections; participation in any clinical study within 30 days preceding the screening visit; insertion of tear duct plugs or ocular surgical procedures within 30 days prior to screening; initiation or modification of systemic medications (e.g., antihistamines, antidepressants, antipsychotics, and benzodiazepines) within 30 days of screening; use of contact lenses within 1 week of the screening visit; ocular surgery within 6 months of screening; or initiation of any topical ocular therapy within 2 weeks before the screening visit.

### Statistical methodology

Linear and logistic regression models with random effects were applied to account for potential correlations between repeated measures from the same patient. The random-effects approach modeled each participant’s influence as a random factor, accommodating within-subject dependency. Additionally, multinomial regression models with clustered robust standard errors were estimated to further address intrasubject correlations. Comparisons of patient characteristics across assessment times (paired samples) were conducted using Cochran’s Q-test. A 5% significance level was adopted for all analyses. Statistical analyses were performed using SPSS version 20.0 and STATA version 17^(11^,^12)^.

## RESULTS

A total of 26 participants (52 eyes) completed the 28-day study protocol. Four participants were lost to follow-up. The discrepancy between the number of eyes assessed in the Schirmer test and the total sample was due to missing data (Table 1).

**Table 1:** Characteristics of the patients and eyes

Patients	
Gender, n (%)	
Female	22/26 (84.6)
Male	4/26 (15.4)
Age (yr)	
Mean ± *SD*	60.0 ± 11.2
Median (IQR)	61.0 (50.8–69.0)
*N*	26
Race, n (%)	
White	20/26 (76.9)
More than one race	4/26 (15.4)
Black	1/26 (3.8)
Asian	1/26 (3.8)
Eyes	
Schirmer test	
Mean ± *SD*	12.8 ± 8.5
Median (IQR)	11.5 (6.8–17.3)
*N*	46

### Characteristics of the patients and eyes

As summarized in table 1, the majority of participants were female (84.6%), with a mean age of 60.0 yr (standard deviation [SD]=11.2 yr). The mean Schirmer I test score was 12.8 mm (SD=8.5 mm).

### Ocular characteristics and quality of life

Table 2 presents the distributions and mean values at baseline and follow-up. Notable improvements were observed across several clinical parameters. VA and TFBUT showed mean increases after 28 days of treatment. The proportion of eyes with TFBUT ≥10 s increased from 25.0% at baseline to 63.5% at follow-up. The proportion of eyes with a corneal staining score of 0 (indicating no epithelial damage) rose from 23.1% to 69.2%. Conjunctival staining scores of 2 and 3 (indicative of moderate to severe damage) decreased from 11.5% to 3.8% and from 5.8% to 0.0%, respectively. The percentage of eyes with a meibomian gland score of 0 (*no dysfunction*) increased from 3.8% to 30.8%, while the percentage with a score of 2 (*moderate dysfunction*) decreased from 46.2% to 21.2%. These findings suggest a clinically meaningful improvement in ocular surface integrity following treatment with the microemulsion eye drops.

**Table 2:** Summary measures of ocular variables by time of assessment

	Evaluation moment		p-value
	Initial day	28 days after	
Eyes			
VA, *N*; mean ± SD	52; 79.87 ± 8.64	52; 84.06 ± 6.52	<0.001^a^
TFBUT, *N*; mean ± SD	47 6.26 ± 5.09	45; 10.04 ± 3.45	<0.001^a^
TFBUT classification, n (%)			0.001^b^
≥10 s	13/52 (25.0)	33/52 (63.5)	
<10 s	39/52 (75.0)	19/52 (36.5)	
Corneal score, n (%)			<0.001^c^
0	12/52 (23.1)	36/52 (69.2)	
1	29/52 (55.8)	12/52 (23.1)	
2	10/52 (19.2)	3/52 (5.8)	
3	1/52 (1.9)	1/52 (1.9)	
Conjunctival score, n (%)			<0.001^d^
0	16/52 (30.8)	23/52 (44.2)	
1	27/52 (51.9)	27/52 (51.9)	
2	6/52 (11.5)	2/52 (3.8)	
3	3/52 (5.8)	0/52 (0.0)	
Alterations in Meibomius glands, n (%)			0.002^c^
0	2/52 (3.8)	16/52 (30.8)	
1	26/52 (50.0)	25/52 (48.1)	
2	24/52 (46.2)	11/52 (21.2)	

Ordered logit proportional odds test: corneal score (p=0.266); meibomian gland alterations (p=0.087).

a Linear model with random effects.

b Logistic model with random effects.

c Ordered logit with clustered robust standard error.

d Multinomial model with clustered robust standard error.

According to table 3, statistically significant differences were observed in both OSDI scores (p<0.001) and total EQ-5D-5L scores (p<0.001) across the assessment periods. The distribution of OSDI scores also differed significantly over time (p<0.001). Specifically, the mean OSDI score was highest during the initial assessment and decreased significantly in the subsequent assessments, which showed similar values. In contrast, the mean total EQ-5D-5L score was lowest at the initial assessment and increased in the later assessments, which were comparable, indicating an overall improvement in quality of life. Furthermore, the proportion of participants with OSDI scores ≤12 increased markedly from 0.0% at baseline to 50.0% at the later time points.

**Table 3:** OSDI and EQ5-D5-L5 scores over time

	Evaluation moment			p-value
	Initial day (n=26)	14 days after (n=26)	28 days after (n=26)	
Individuals				
OSDI, *N*; mean ± SD	47.12 ± 13.26^c^	16.38 ± 13.97^d^	16.27 ± 12.01^d^	<0.001^a^
OSDI’s classification, n (%)				<0.001^b^
≤12	0/26 (0.0)	13/26 (50.0)	13/26 (50.0)	
≥13	26/26 (100.0)	13/26 (50.0)	13/26 (50.0)	
EQ5-D5-L5 total, N; mean ± SD	0.73 ± 0.25^d^	0.85 ± 0.14^c^	0.84 ± 0.14^c^	<0.001^a^
EQ5-D5-L5 VAS, N; mean ± SD	74.04 ± 18.33	76.54 ± 17.13	75.92 ± 15.63	0.591^a^

a Linear model.

b Cochran’s Q-test with random effects.

c,d Different means according to multiple comparisons with the Bonferroni correction.

### Tolerance to the study of eye drops and adverse events

As presented in table 4, there was no significant difference in pain scores reported between assessment points (p=0.998), suggesting good overall tolerability of the treatment.

**Table 4:** Distribution of eye pain scores

	Evaluation moment		p-value
	14 days after	28 days after	
Pain, n(%)			0.998
No pain	50/52 (96.2)	50/52 (96.2)	
Mild pain	2/52 (3.8)	0/52 (0.0)	
Severe pain	0/52 (0.0)	2/52 (3.8)	

p-value from multinomial model with clustered robust standard error.

According to table 5, mild, transient stinging sensations were reported in 34.6% of treated eyes (95% confidence interval [95% CI], 22.0-49.1). No serious adverse events were recorded.

**Table 5:** Distribution of adverse events in the eyes

Adverse event	n (%)	95% CI
No adverse events	33/52 (63.5)	49.0–76.4
Eye sting	18/52 (34.6)	22.0–49.1
Itching	1/52 (1.9)	0.05–10.3

95% CI, 95%.

Confidence interval.

## DISCUSSION

An increase in the percentage of patients with TFBUT ≥10 s was observed between the first and second evaluations (25.0%-63.5%), suggesting that the eye drops used in this study may improve tear-film stability and reduce evaporation. Similar improvement in TFBUT was reported by Maity et al.^(13)^ with the use of artificial tears containing propylene glycol.

A significant increase was also observed in the percentage of patients with a corneal staining score of 0, rising from 23.1% on Day 1 to 69.2% on Day 28. This improvement indicates enhanced tear-film function, reinforcing its role as a protective barrier and reducing damage to the corneal epithelium. Regarding the conjunctival score, reductions were observed in the proportions of patients with scores of two (11.5%-3.8%) and 3 (5.8%-0.0%) over the 28-day period. A literature review by Srinivasan et al.^(14)^ demonstrated that propylene glycol-based artificial tears provide optimal protection and lubrication to the ocular sur face, promoting the healing of corneal damage associated with DED.

Statistically significant differences in OSDI scores were observed across the assessment time points (p<0.001). The mean OSDI score decreased from 47.12 (SD ± 13.26) at baseline to 16.27 (SD ± 12.01) after 28 days of treatment. Additionally, there was a significant shift in the distribution of OSDI scores (p<0.001). While the mean OSDI score was notably higher at the initial assessment, it remained consistent at subsequent evaluations. The proportion of patients with OSDI scores ≤12 increased from 0.0% at baseline to 50.0% after treatment. These findings are consistent with those of Maharana et al.^(15)^, who reported that propylene gly col-based artificial tears significantly improved OSDI scores after 28 days of use.

The safety and tolerability of the treatment were assessed using a pain score. No significant differences were observed in the distribution of pain scores during treatment, indicating good tolerability. Furthermore, patients reported an improvement in quality of life. These findings are supported by a prospective study by Torkildsen et al.^(10)^, which confirmed the safety and tolerability of the same ophthalmic lubricant eye drops.

In conclusion, the eye drops demonstrated clinical effectiveness in the management of DED. The observed increase in TFBUT and improvement in corneal scores suggest enhanced tear-film stability and reduced evaporation. The marked improvement in OSDI scores reflects substantial symptom relief. Moreover, the treatment was found to be safe and well tolerated, providing protection to the ocular surface and contributing to the reduction of inflammation and discomfort.

## Data Availability

The contents underlying the research text are included in the manuscript
